# Autologous fecal transplantation from a lean state potentiates caloric restriction effects on body weight and adiposity in obese mice

**DOI:** 10.1038/s41598-020-64961-x

**Published:** 2020-06-10

**Authors:** Patricia Pérez-Matute, María Íñiguez, María de Toro, Emma Recio-Fernández, José A. Oteo

**Affiliations:** 1 Infectious Diseases, Microbiota and Metabolism Unit. Infectious Diseases Department, Center for Biomedical Research of La Rioja (CIBIR), Logroño, La Rioja Spain; 2 Genomics & Bioinformatics Core Facility. CIBIR, Logroño, La Rioja Spain; 3 Infectious Diseases Department. Hospital San Pedro, Logroño, La Rioja Spain

**Keywords:** Obesity, Fat metabolism, Metagenomics

## Abstract

Autologous fecal transplantation (FT-A) emerges as a promising strategy to modulate gut microbiota with minimal side effects since individual´s own feces are transplanted. With the premise of improving obesity and its associated disorders, we investigated if fecal microbiota transplantation (FMT), heterologous and autologous, potentiates the effects of a moderate caloric restriction (CR) in high-fat diet (HFD)-induced obese mice. Mice were randomized into control, HFD, CR (12 weeks on HFD and 6 weeks under CR), FT-H (similar to CR and FMT carried out with feces from controls, weeks 17 & 18), and FT-A (administration of their own feces before developing obesity at weeks 17 & 18). Our study demonstrated that FMT, and, especially, FT-A potentiates the effects of a moderate CR on weight loss and adiposity in the short term, by decreasing feed efficiency and increasing adipose tissue lipolysis. Although FT-A produced a significant increase in bacterial richness/diversity, FMT did not significantly modify gut microbiota composition compared to the CR at phyla and bacteria genera levels, and only significant increases in *Bifidobacterium* and *Blautia* genera were observed. These results could suggest that other mechanisms different from bacterial microbiota engraftment participates in these beneficial effects. Thus, FT-A represents a very positive synergetic approach for obese patients that do not respond well to moderate restrictive diets.

## Introduction

Obesity is a chronic disease characterized by an excess of body weight with excessive/abnormal fat accumulation. It is one of the leading causes of morbidity and mortality nowadays with important economic and social costs as it is associated with a large number of health problems including dyslipidemia, type 2 diabetes, cardiovascular diseases, non-alcoholic fatty liver and certain types of cancer^1–3^. Obesity is the result of physiological/endocrine, behavioral and environmental factors. In this sense, the combination of excessive calorie consumption and sedentary practices are the main drivers of weight gain, and, therefore, responsible for the rapid acceleration of the obesity epidemic worldwide^4^. Lifestyle modification programs promoting a negative energy balance represent a first line of therapy for obesity management^5,6^. However, these classical lifestyle interventions including improvements in diet and physical activity induce heterogeneous responses in the overweight/obese subjects ranging from resistance to reduce body fat mass to even unsuccessful long-term weight loss maintenance^5,7,8^. Genetics and epigenetics signatures (SNPs, DNA methylation, histone modifications, gene expression profiles, miRNA expression etc…) have been found to modulate the effects of nutritional treatments on weight loss and weight regain (reviewed by Ramos-Lopez *et al*., 2017)^8^. Gut microbiota emerges as an interesting factor that contributes to the onset of obesity and involves in long-term successful weight loss strategies^9–15^.

The pivotal role of microbiota in health and disease has generated much interest in the past decade. Microbiota is defined as a collection of microorganisms inhabiting a specific environment and includes bacteria, archaea, viruses and some unicellular eukaryotes. Gut microbiota is considered an organ able to perform complex functions and to produce different metabolites, which are capable of interacting with the host via direct or indirect mechanisms, and, thereby, influencing host metabolism^16^. Thus, the old model based on direct interactions between environmental factors and genetic variations of individuals (nutrigenetic, nutrigenomic, and nutriepigenetic interactions) has changed taking into consideration the relationships among the gut microbiota and the host^17^. A large body of evidence supports that gut-microbiota-based therapies (antibiotics, probiotics, prebiotics, symbiotics) can be an effective approach to modulate host metabolism, and, therefore, to treat obesity and other metabolic disorders^15,18^.

Fecal microbiota transplantation (FMT) is an interesting option to modify gut microbiota as it has been associated with improved clinical outcomes in recurrent *Clostridioides difficile* infection^19–22^. However, the potential usage of FMT in other microbiota-associated conditions different from *C. difficile* such as inflammatory bowel disease, metabolic syndrome or obesity is still under investigation^23–27^. Fecal microbiota transplantation has also logistical challenges such as FMT standardization, including donor selection, FMT material preparation and administration routes along with proper regulation^28,29^. In this context, autologous transplant or autotransplant (transplantation of the individual´s own feces before developing the disease) emerges as a well-tolerated, safe and more appropriate approach from an ethical point of view. In fact, autotransplant theoretically has a more desirable safety profile than heterologous fecal transplants because the feces come from the same patient in a healthy state and, therefore, will minimize the risk of exposure to potentially pathogenic microorganisms not previously encountered by the patient. With the premise of improving obesity and its associated disorders, we have investigated if fecal transplantation, heterologous and autologous, potentiates the effects of a moderate caloric restriction (CR) on body weight gain and adiposity in obese mice. For the autologous transplantation, each animal received their own faeces but collected before these mice developed obesity. To our knowledge, there is no experience in this regard.

## Results

### Effects of fecal transplantation (heterologous and autologous) on body weight, feed efficiency, adipose tissue and liver weight

**Figure 1:**
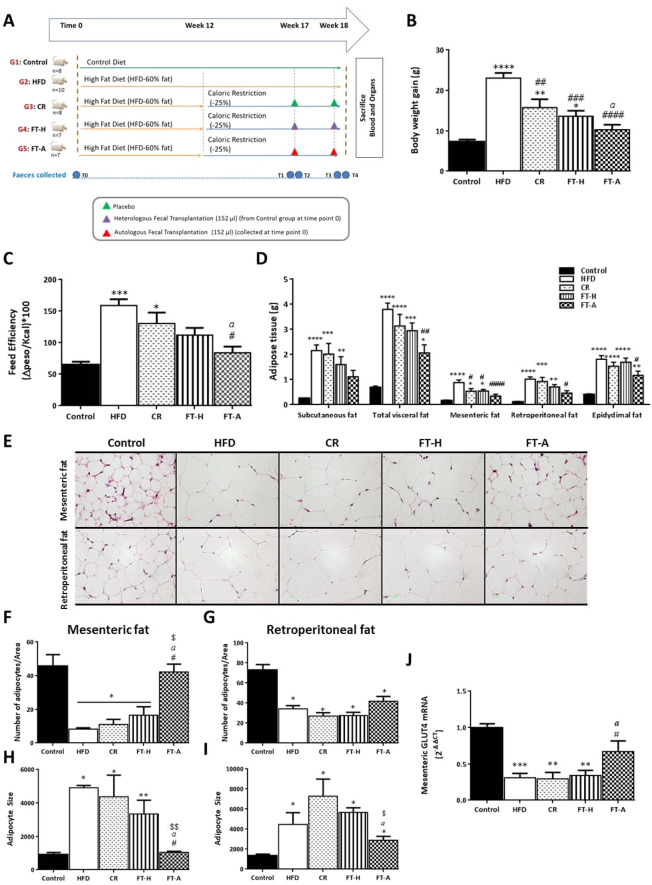
Schematic design of the experiment and effects of fecal microbiota transplantation (FMT) on body weight gain, feed efficiency and adiposity. (**A**) Schematic design of the experiment. (**B**) Body weight gain of mice fed with a control or HFD and submitted to CR with and without FMT. (**C**) Metabolic efficiency ratio representing the body weight gain relative to energy intake among the groups. (**D**) Effects of CR and FMT on subcutaneous, mesenteric, retroperitoneal, epididymal and total visceral fat weights in control and HFD-induced obese mice. (**E**) Representative H&E staining of mesenteric and retroperitoneal fat depots. Scale bar, 10 µm. (**F-I**) Quantification of number and size of adipocytes per area (627.42 × 468.29 µm). **(J**) Effects of CR and FMT on mesenteric GLUT4 mRNA levels. Data are expressed as mean ± SEM of at least 8 animals per group. *p < 0.05, **p < 0.01, ***p < 0.001, ****p < 0.0001 *vs* Control; ^#^*p* < 0.05, ^##^*p* < 0.01, ^###^*p* < 0.001, ^####^*p* < 0.0001 *vs* HFD, ^*a*^*p* < 0.05 *vs* CR^, $^*p* < 0.05, ^$$^*p* < 0.01 *vs* FT-H.

The increased body weight gain induced by a HFD (p < 0.0001 *vs*. control) was lower in those animals under a moderate caloric restriction for 6 weeks (p < 0.01 *vs*. control and *vs*. HFD). This lower body weight gain observed in animals under CR was more evident in the fecal-transplanted mice and especially in the FT-A group as significant differences were observed among the FT-A mice and the CR group (p < 0.0001 FT-A *vs*. HFD and p < 0.05 *vs*. CR). No differences were observed in body weight gain among the FT-A group and the controls at the end of the experiment (Fig. 1B). The feed efficiency ratio, representing the body weight gain relative to energy intake in calories, was significantly higher in the HFD group (p < 0.001 *vs*. control). A moderate caloric restriction was able to partially impair such increase (p < 0.05 *vs*. control). FT-A mice showed a lower feed efficiency ratio in comparison with the HFD and also compared to the CR group (p < 0.05 *vs*. HFD and CR), although no statistical differences were observed among FT-A and FT-H animals (Fig. 1C). No differences were observed in food intake (expressed as grams ingested per day or calories ingested per day per animal) among the three groups under CR (Supplementary table 1). A similar pattern was observed in adipose tissue; thus, the ingestion of a HFD for 18 weeks was accompanied by a significant increase in subcutaneous fat and also in total visceral fat and in each of the fat pads collected (p < 0.0001 *vs*. control) (Fig. 1D) whereas fecal transplantation, especially the FT-A group, showed a significant lower size of total visceral fat compared to the HFD group (p < 0.01 *vs*. HFD), being more evident in the mesenteric and retroperitoneal fat depots (p < 0.0001 and p < 0.05 *vs*. HFD respectively). In addition, no significant differences were observed in the weight of these fat depots in the FT-A group in comparison with the controls and also compared to the FT-H animals (Fig. 1D). Histological examination of mesenteric and retroperitoneal adipose tissues showed fewer and bigger adipocytes in HFD-fed mice (Fig. 1E–I). FT-A mice showed a significant increase in the number of mesenteric adipocytes (p < 0.05 *vs*. HFD and *vs*. CR) with no statistical differences when compared to the controls. Thus, the size of the mesenteric adipocytes in FT-A mice were significantly smaller than those observed in the HFD group, and also in comparison with the CR mice (p < 0.05) and the FT-H (p < 0.01) with no differences when compared to the controls (Fig. 1F–H). A similar pattern was observed in retroperitoneal adipose tissue although less significant than in the mesenteric depot (Fig. 1G–I). Antibiotic (neomycin + ampicillin) treatment for 6 days prior fecal transplants largely abolishes the effects of FT (both heterologous and autologous) on body weight gain and adipose tissue size (Supplementary figure 1A-B). A significant increase was observed in liver size and transaminases plasma levels after the ingestion of a HFD. Six weeks under CR was able to counteract such increase independently of the fecal transplantations carried out (Supplementary figure 2A-B).

### Effects of Fecal Transplantation (Heterologous and Autologous) on glucose metabolism, triglyceride (TG) serum levels and bacterial translocation

As observed in Table 1, the ingestion of a HFD for 18 weeks induced a significant increase in glucose and insulin serum levels as well as in the insulin resistance index, the HOMA index, compared to the control animals (p < 0.05). A moderate CR diet for 6 weeks did not result in significant improvements in these parameters. However, FT-mice (both heterologous and autologous FT-mice) showed a significant decrease in all parameters (p < 0.05 *vs*. HFD and p < 0.05 *vs*. CR). No differences were observed on insulin and the HOMA index when comparing the FT-H or FT-A groups with the controls (Table 1). The ingestion of a HFD resulted in a significant reduction of the mRNA levels of GLUT-4 in mesenteric fat (Fig. 1J), however, this decrease was not counteracted by the CR nor FT-H, whereas a significant increase was observed in FT-A (p = 0.05 *vs*. HFD and CR). TG serum levels were significantly increased after the ingestion of a HFD for 18 weeks (p < 0.05) and FT were able to significantly impair such increase, being especially evident in the FT-A group (p < 0.05 *vs*. HFD and *vs*. CR) (Table 1). Our results showed that HFD mice presented a significant increase in LBP plasma levels and CR was able to reduce such increase, independently of the fecal transplantations (Supplementary figure 5).

**Table 1:** Glucose metabolism and serum triglycerides levels upon CR and gut microbiota transplantation.

	Control	HFD	CR	FT-H	FT-A	Kruskal-Wallis test P value
Glucose (mg/dL)	282.0 ± 28.22	424.0 ± 72.30	309.8 ± 12.31	166.0 ± 32.97^#*aa*^	119.5 ± 20.40*^#*a*^	0.004
Insulin (ng/mL)	1.24 ± 0.35	7.41 ± 0.49*	6.02 ± 1.73*	2.41 ± 0.84^#^	1.25 ± 0.36^#*a*^	0.010
HOMA index	22.26 ± 8.17	117.8 ± 0.00*	128.7 ± 40.05*	12.41 ± 5.12^#*a*^	7.22 ± 2.78^#*a*^	0.003
Triglycerides (mg/dL)	86.00 ± 5.19	146 ± 13.25*	112.5 ± 6.78*	99.0 ± 0.57	89.0 ± 3.08^#*a*^	0.003

Data are expressed as mean ± SEM of at least 8 animals per group. *p < 0.05 vs Control; #p < 0.05 vs HFD, ap < 0.05, aap < 0.01 vs CR according to Kruskal-Wallis test followed by Mann Whitney U-tests.

### Fecal Transplantation (Heterologous and Autologous) induces lipolysis in white adipose tissue (WAT)

**Figure 2:**
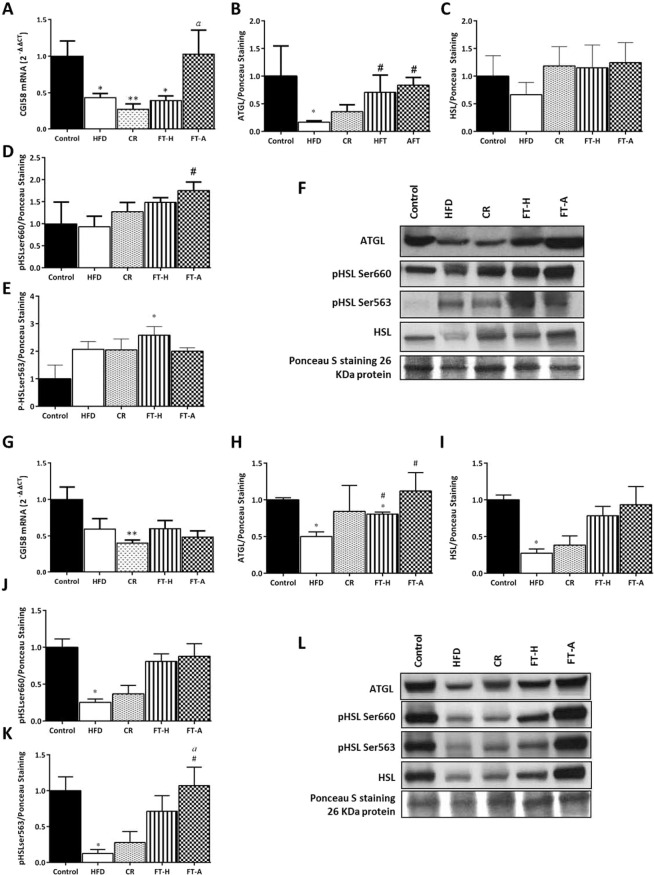
Effects of FMT on lipolysis in adipose tissue. Effects of FMT on (**A**) mRNA levels of ATGL coactivator CGI58, (**B**) total ATGL protein levels, (**C**) total HSL protein levels and **(D, E, F)** HSL phosphorylation in mesenteric fat from control and HFD mice. Effects of FMT on (**G**) mRNA levels of ATGL coactivator CGI58, (**H**) total ATGL protein levels, (**I**) total HSL protein levels and (**J, K, L**) HSL phosphorylation in retroperitoneal fat from control and HFD mice. Blots presented in this figure come from the same part of the same gel/blot. Full-length gels are provided as supplementary figures (Supplementary Figures 6-10). To achieve the number of samples needed for statistical analyses (n = 4), two gels/blots were used and were processed in parallel. Loading control (Ponceau S) has been run in the same blot to normalize the results. Data are expressed as mean ± SEM of 4 animals per group in the western-blot analyses while all animals were included for the quantification of CGI58 mRNA levels (at least 8 animals per group). Data are presented in comparison to the controls considered as 1. *p < 0.05, **p < 0.01, *vs* Control; ^#^*p* < 0.05, ^##^*p* < 0.01, *vs* HFD, ^*a*^*p* < 0.05, ^*aa*^*p* < 0.01 *vs* CR, ^$$^*p* < 0.01 *vs* FT-H.

To understand the molecular mechanisms underlying the antiadiposity effects of FT on white adipose tissue, the expression of the main lipolysis-related enzymes in mesenteric and retroperitoneal adipose tissues was evaluated (Fig. 2). A significant increase in total ATGL protein expression was observed in both transplanted-mice in comparison with the HFD group (p < 0.05) (Figs. 2B,H). Significant higher CGI58 mRNA levels were also observed in the FT-A group in mesenteric adipose tissue (p < 0.05 *vs*. CR) (Fig. 2A). HSL activity is regulated by reversible phosphorylation in serine residues. PKA phosphorylates HSL at Ser 563 and Ser 660, which stimulates HSL activity. In contrast, phosphorylation of HSL at Ser 565 by AMPK prevents activation by PKA, inhibiting lipolysis. Thus, to better elucidate the mechanisms underlying the lipolytic actions of FT, we investigated the effects of FT (both heterologous and autologous) on HSL phosphorylation in Ser 563, Ser 660 and Ser 565 in both mesenteric and retroperitoneal fat pads from four animals per group (Figs. 2F,L). No significant differences were observed on mesenteric HSL protein expression (Fig. 2C–F) whereas a significant increase was observed on HSL phosphorylation in Ser 660 in FT-A mice in mesenteric fat compared to HFD (p < 0.05 *vs*. HFD and p = 0.08 *vs*. CR) (Fig. 2D–F). No significant differences were observed on HSL phosphorylation in Ser 563 (Fig. 2E,F). Concerning retroperitoneal adipose tissue, the significant decrease observed on ATGL after the ingestion of a HFD was counteracted by FT, especially by FT-A at the protein level (p < 0.05 *vs*. HFD for both FT-H and FT-A) (Fig. 2H–L). FT was also able to counteract the lower total HSL protein levels induced by the HFD (Fig. 2I–L), as well as the HFD-induced reduction in HSL phosphorylation in ser660 (it did not reach statistical significance) and ser563 (p < 0.05 FT-A *vs*. HFD and *vs*. CR) (Fig. 2J–L). No statistical differences were observed in HSL phosphorylation in Ser 565 in either mesenteric or retroperitoneal adipose tissue (data not showed).

### Fecal Transplantation (Heterologous and Autologous) increases fatty acid oxidation in liver

**Figure 3:**
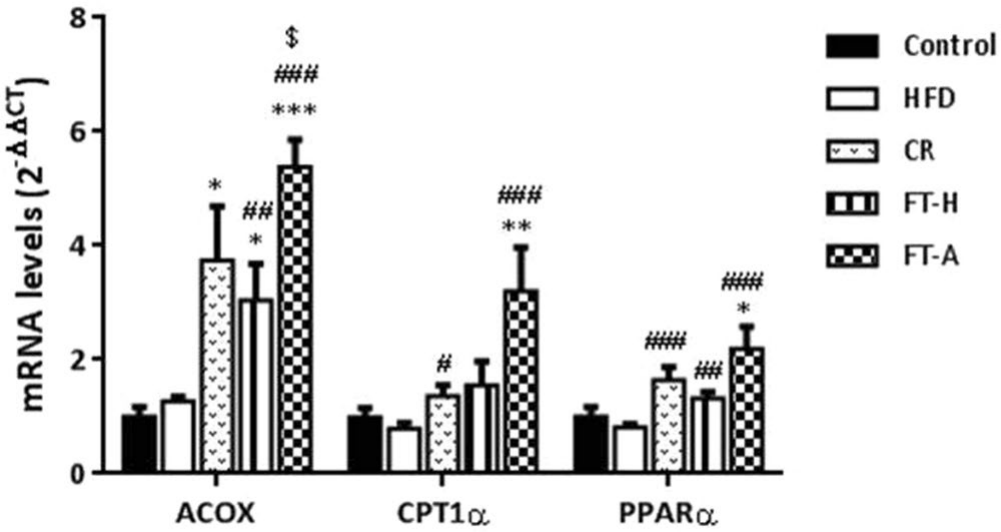
Effects of FMT on fatty acid oxidation in liver. Effects of FMT on hepatic mRNA levels of ACOX, CPT1α and PPARα in control and HFD mice. Data are expressed as mean ± SEM of at least 8 animals per group. *p < 0.05, ***p < 0.001, *vs* Control; ^#^*p* < 0.05, ^##^*p* < 0.01, ^###^*p* < 0.001 *vs* HFD and ^$^*p* < 0.05 *vs* FT-H.

Although no significant differences were observed in the mRNA levels of PPARα, ACOX or CPT1α in adipose tissue due to the fecal transplantations (data not showed), a significant increase was observed in the hepatic expression of all of these genes in the FT-A group compared to the HFD animals (Fig. 3). Six weeks under a moderate caloric restriction and the heterologous fecal transplantation were also accompanied by significant increases in the mRNA levels of ACOX, CPT1α and PPARα in liver, however, the induction of these beta-oxidation genes was more potent in those animals treated with autologous fecal transplants (p < 0.001 *vs*. HFD and *vs*. CR) (Fig. 3). In addition, significant differences were observed in the mRNA levels of ACOX when comparing both types of transplants (p < 0.05 FT-H *vs*. FT-A).

### Effects of Fecal Transplantation (Heterologous and Autologous) on bacterial diversity/richness

**Figure 4:**
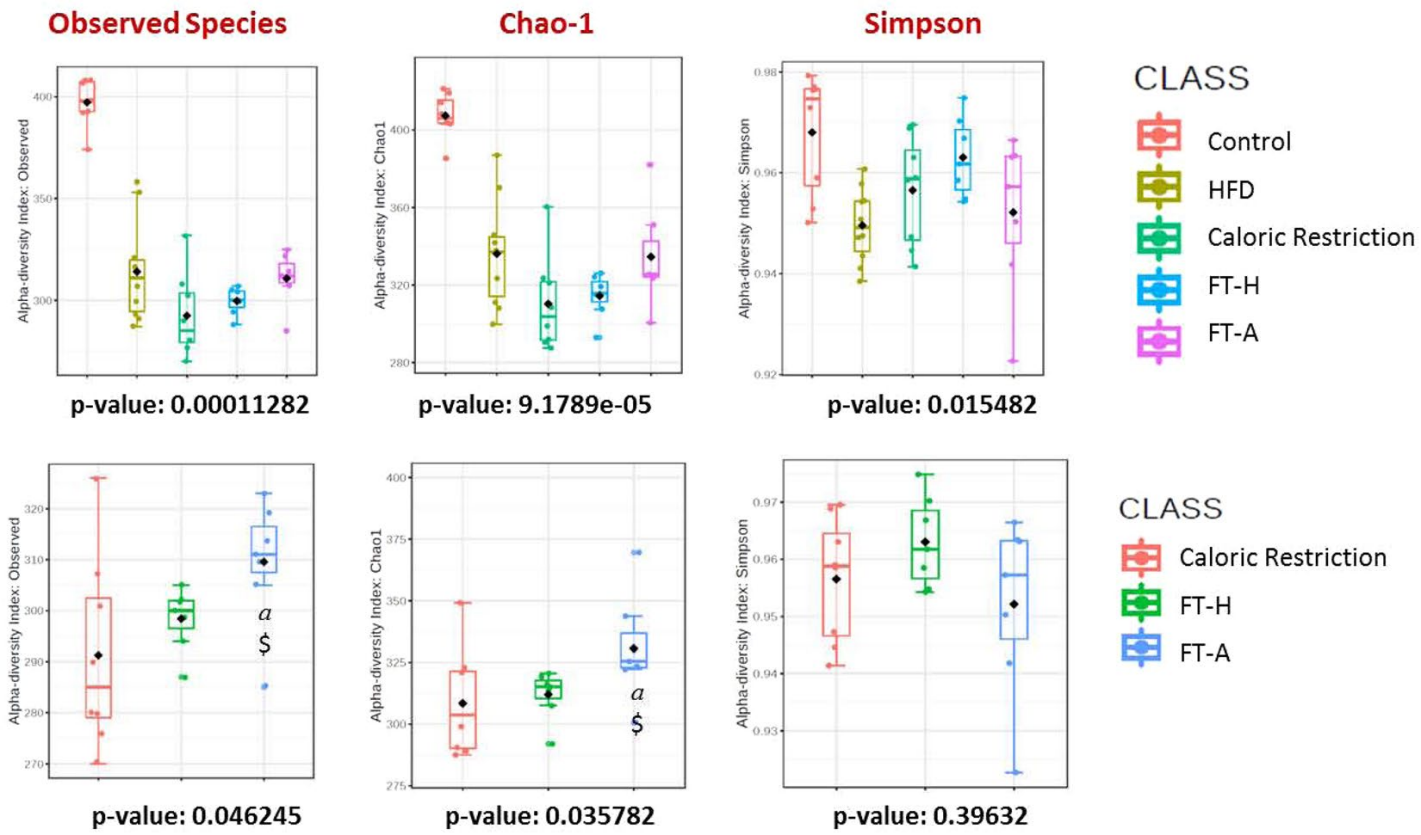
α-diversity in control and HFD mice: effects of Fecal Transplantation. Data are expressed as mean ± SEM of at least 8 animals per group. ^*a*^*p* < 0.05 *vs* CR, ^$^*p* < 0.05 *vs* FT-H.

**Figure 5:**
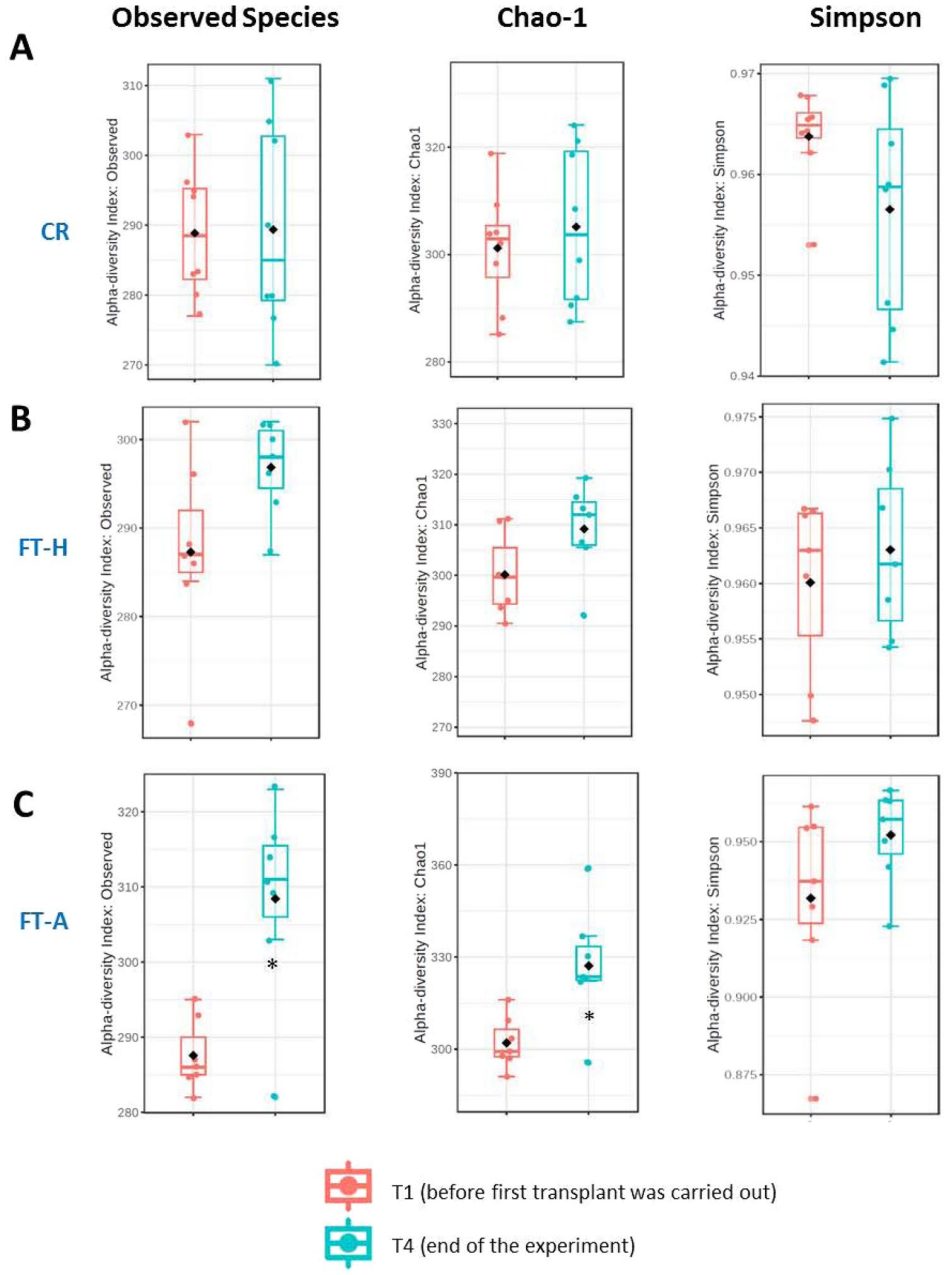
Longitudinal study of α-diversity in mice under CR with or without FMT. Effects of caloric restriction (**A**), heterologous FMT (**B**) and autologous FMT (**C**) on Observed Species, Chao-1 and Simpson indexes. Data are expressed as mean ± SEM of at least 8 animals per group. *p < 0.05 *vs* Control.

Two indexes of bacterial richness (Observed species and Chao-1) and one of dominance (Simpson index) have been evaluated. At the end of the experimental period, a significant decrease in bacterial richness was observed after the ingestion of a HFD. This was not counteracted by 6 weeks on CR (Fig. 4). A tendency to improve such decrease was observed in the FT-A group, being especially significant with the Observed species index (p = 0.05 *vs*. CR and p < 0.05 *vs*. FT-H) and the Chao-Index (p < 0.05 *vs*. CR and FT-H). Interestingly, when α-diversity was compared among T1 (24 hours before the first transplant was carried out) and the end of the experimental period (T4), no differences were observed in the CR group or in the FT-H groups (Fig. 5A,B). However, a significant increase was observed in α-diversity in the FT-A group (p < 0.05-p = 0.01 for Observed species and the Chao-1 indexes respectively), while no differences were found in the Simpson index (Fig. 5C).

### Effects of Fecal Transplantation (Heterologous and Autologous) on microbial communities’ composition

**Figure 6:**
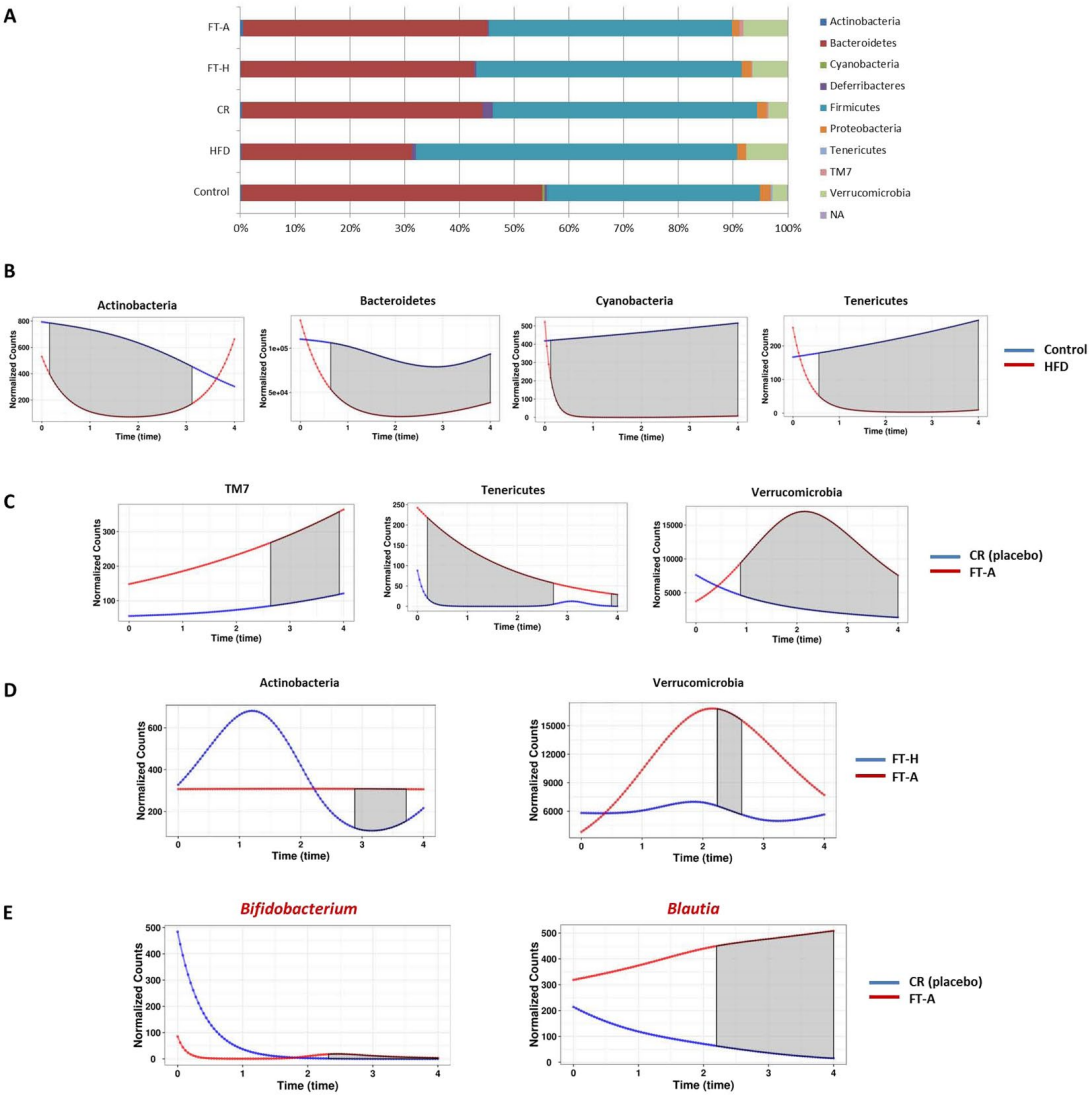
Effects of FMT on gut microbiota composition at phylum and genera level. (**A**) Relative abundance of major bacterial phyla present in gut expressed as percentage at the end of the experimental period (T = 4) (**B**) Temporal dynamics of gut phyla that resulted statistical significant comparing the control and HFD mice. (**C**) Temporal dynamics of gut phyla that resulted statistical significant comparing the CR and autologous transplanted mice (CR *vs*. FT-A). (**D**) Temporal dynamics of gut phyla that resulted statistical significant when comparing the heterologous and autologous transplanted mice (FT-H *vs*. FT-A). (**E**) Temporal dynamics of gut bacterial genera that resulted statistical significant comparing the CR and autologous transplanted mice (CR *vs*. FT-A). The shaded area in gray means the time interval where the corresponding taxonomic group is significantly different among the two groups of mice and was calculated by the MetaLonDA R-package.

Concerning GM composition (differential abundance among groups of animals), the analysis carried out at the end of the experimental period (T4) revealed that the most abundant phyla in all groups were Bacteroidetes and Firmicutes, representing around of the 90% of phyla in stools (Fig. 6A). It is interesting to note that the increase observed in Firmicutes and, on the contrary, the decrease observed in Bacteroidetes in the HFD group was partially restored by the CR, and independently of the type of transplant carried out (Fig. 6A).

When comparing the five groups using a classical univariate analyses, five phyla resulted statistical different: Cyanobacteria, Firmicutes, Tenericutes, TM7 and Actinobacteria (Table 2). However, the differences observed in TM7 and Actinobacteria abundance could be due to the slight increase observed in FT-A in comparison with the other groups (data not showed), although it did not reach statistical significances when compared to the CR group. Temporal dynamics at phylum level were also analyzed. The ingestion of a HFD induced a significant decrease in the presence of Actinobacteria, Bacteroidetes, Cyanobacteria and Tenericutes along the experimental period (p = 0.0001-p.003) (Fig. 6B). When studying the temporal dynamics among the FT-A group against their placebo (CR), significant increases were observed on TM7, Tenericutes and Verrucomicrobia in FT-A animals (p = 0.0006-p = 0.45) (Fig. 6C). Of interest, the increased abundance of TM7 in FT-A resulted significant only after applying the first transplant. No statistical differences were observed among the FT-H and FT-A groups in the longitudinal study at phyla level. Only slight differences were observed in Actinobacteria (p = 0.047) and Verrucomicrobia (p = 0.00), although these differences disappeared at the end of the experimental period, T4 (Fig. 6D).

**Table 2:** Statistical comparisons of abundance of the major phyla present in gut when control mice were compared with those fed with a HFD, submitted or not to a moderate caloric restriction and under fecal transplants (heterologous and autologous).

PHYLA	FDR	HFD vs. CONTROL	CR vs. CONTROL
Cyanobacteria	3.35E^−04^		
Firmicutes	4.31E^−04^		
Tenericutes	4.42E^−04^		
TM7	0.010941	No effects	No effects
Actinobacteria	0.020097	No effects	No effects

A false discovery rate (FDR) < 0.05 was considered significant. FDR was obtained comparing the five groups using Kruskal-Wallis test for univariate comparison and the Benjamini-Hochberg approach.

At genus level, when comparing the five groups of animals at the end of the experimental period (T4), 19 genera resulted statistical significant among the groups (Table 3). Six weeks under caloric restriction was able to impair the HFD effects on the abundance of *Lactococcus*, *Anaerotruncus*, *Oscillospira*, *Streptococcus*, *Bilophila* and *Ruminococcus* (Table 3). Fecal transplants did not translate into substantial changes at genus level in comparison with the effects of caloric restriction and only significant increases were observed in *Bifidobacterium* and *Blautia* genera (Table 3). In fact, when comparing the three groups under CR, only significant differences were observed on these two bacterial genera: *Blautia* and *Bifidobacterium* (FDR: 0.0438). Thus, higher abundance of *Blautia* was observed in both FT animals in comparison to the placebo group (CR) and also higher presence of *Bifidobacterium* was observed in both FT compared to CR, being more evident in the FT-A mice (Supplementary figure 3). Temporal dynamics at genera level were also analyzed. The increase observed in *Bifidobacterium* and *Blautia* was observed after the first transplant, as can be observed in Fig. 6E. No significant differences were observed among FT-H and FT-A at bacterial genus level.

**Table 3:** Statistical comparisons of abundance of the major genera present in gut when control mice were compared with those fed with a HFD, submitted or not to a moderate caloric restriction and under fecal transplants (heterologous and autologous).

Genera	Phylum	FDR	HFD vs. Control	CR vs. Control	Effects of Transplants
*Lactococcus*	Firmicutes	8.4002e-05			No
*Prevotella*	Bacteroidetes	8.4002e-05			No
*Clostridium*	Firmicutes	9.8581e-05			No
*Bifidobacterium*	Actinobacteria	0.00032326			Yes* Increase
*Adlercreutzia*	Actinobacteria	0.00032326			No
*Anaeroplasma*	Firmicutes	0.00032326			No
*Anaerotruncus*	Firmicutes	0.00038042			No
*Oscillospira*	Firmicutes	0.00038042			No
*Sutterella*	Proteobacteria	0.00045744			No
*Streptococcus*	Firmicutes	0.00045744			No
*Bilophila*	Proteobacteria	0.00049813			No
*Ruminococcus*	Firmicutes	0.00053553			No
*Odoribacter*	Bacteroidetes	0.00061392	—		No
*Parabacteroides*	Bacteroidetes	0.002023			No
*Dehalobacterium*	Firmicutes	0.0027531			No
*Blautia*	Firmicutes	0.0043595			Yes* Increase
*Bacteroides*	Bacteroidetes	0.0076317			No
*Dorea*	Firmicutes	0.017783			No
*Lactobacillus*	Firmicutes	0.033351		—	No

A false discovery rate (FDR) < 0.05 was considered significant. FDR was obtained comparing the five groups using Kruskal Wallist test and the Benjamini-Hochberg approach.

Taking into account Bray-Curtis index, all groups clustered together at the beginning of the experimental period (Supplementary figure 4). The ingestion of a HFD was crucial. In fact, the controls and the other groups (HFD, CR and both transplanted mice) were represented in two different clusters (PERMANOVA r^2^: 0.59426, p < 0.001) at T1. At the end of the experimental period (T4), the same pattern was observed, although the clusters seem to be more homogenous (PERMANOVA r^2^: 0.53974, p < 0.001) and the FT-A was represented inside the others (Supplementary figure 4). These results were plotted according to the first two principle components and the clustering of samples was represented accounting for 54.5% of total variation (Component 1 = 41.5% and Component 2 = 13%).

## Discussion

Autologous fecal transplantation emerges as a promising strategy to modulate gut microbiota with minimal long-term side effects since individual´s own feces are transplanted avoiding the risks of donor resistance genes and donor-recipient mismatches. Several recent studies have confirmed the potential for autologous fecal transplantation for remediation of gut microbiota after antibiotic treatments^30–32^. Here, we have developed a mouse model of autologous transplantation in which each animal received their own feces but collected before the mice were obese. The extrapolation of this model into humans will require the creation of fecal banks, along with the proper legislation and safety controls.

Our main finding is that only two autologous fecal transplantations were able to potentiate the effects of a moderate energy restriction on weight loss and adiposity in the short term, possibly by decreasing feed efficiency and by increasing adipose tissue lipolysis and hepatic fatty acid oxidation. Although heterologous fecal transplantation also showed positive effects, the majority of the results suggested that autotransplant is apparently more potent than CR alone and also than the FT-H group as these animals better mimic the physiology of mice fed with a standard diet (control animals).

The lower body weight gain observed in the FT-A mice seems to be related to the ability of the autologous fecal transplantation to reduce the amount of energy absorbed from food (feed efficiency), similarly to the results obtained by Lai *et al*., (2018) that demonstrated that HFD-fed mice receiving FT from control-exercised donors showed remarkably reduced food efficacy^33^. In this context, a vast majority of studies have demonstrated that a high Firmicutes/Bacteroidetes ratio is associated to increased capacity for harvesting energy from diet, as observed in obese subjects/animals^15,34^. Similarly, our HFD animals exhibited a higher proportion of Firmicutes and lower abundance of Bacteroidetes in comparison with those fed with a control/standard diet, which could explain the increased feed efficiency observed in these animals. Energy restriction was able to significantly counteract the increase observed in Firmicutes with no significant effects on Bacteroidetes and feed efficiency. Fecal transplantations, both heterologous and autologous, did not exert any significant effects on the abundance of these phyla suggesting that the lower feed efficiency observed in these animals are independent of the abundance of Firmicutes and Bacteroidetes phyla, which contrasts with other studies that observed that FT intervention mitigated the HFD-disrupted gut microbiota at phylum level^35^. These differences could be explained by the different methodology used in both studies (FT once a day for 8 weeks *vs*. once per week for only two weeks in our study). It seems that longer and more frequent transplants are needed in order to induce changes in gut microbiota at phylum level. However, since only two FT are able to induce in the short term a significant decrease in feed efficiency and a lower body weight and adiposity; this is a more interesting approach to extrapolate into humans.

In the same line, a lower richness of microbiome gene content and taxa has been tightly linked to a higher feed efficiency^36^. Fecal microbiota transplantation in mice was associated with increased bacterial richness^37^. Similarly, our study has demonstrated that autologous fecal transplantation exerted a significant increase in α-diversity in the longitudinal analysis, which could explain, at least in part, the lower feed efficiency observed in these animals at the end of the experimental period and, therefore, the lower body weight gain. These results could suggest that the anti-obesity effects of autologous fecal transplantation are more related to changes in bacterial diversity rather than in wide phylum levels. In addition, FT and especially autologous fecal transplantation significantly improved insulin resistance despite a HFD, as previously demonstrated in individuals with metabolic syndrome^26^ and also in mice^35^, which reinforces the benefits of FT on obesity and associated disorders. These positive results observed in glucose metabolism could be secondary, at least in part, to the increased expression of the facilitated glucose transporter member 4 (GLUT4) in adipose tissue^38^, although more studies are needed to deeply investigate the underlying mechanisms.

The lower body weight gain observed in FT-A mice was also accompanied by a lower adiposity, being especially evident in total visceral fat and specifically in the mesenteric and retroperitoneal fat pads. These are the fat depots strongly linked to insulin resistance, type 2 diabetes, hypertension, and dyslipidemia, suggesting that the reduction of these adipose tissues could also contribute to the protective actions observed on insulin resistance and other metabolic alterations. Elucidations of molecular mechanisms that favor adipose tissue decline such as increased lipolysis and/or β-oxidation are of interest. In fact, the significant increase observed in lipolysis in both fat depots could explain the lower weight of adipose tissue in general and also the lower size of adipocytes. Lipolysis is a complex process that is highly regulated and involves the coordinated participation of several lipid droplet proteins and also several lipases such as adipose triglyceride lipase (ATGL/desnutrin), hormone sensitive lipase (HSL), and monoacylglycerol lipase^39^. A significant increase in total ATGL protein expression was observed in both fat depots in FT-mice in comparison with the HFD group. Lipase activity of ATGL largely depends on its coactivation by comparative gene identification 58 (CGI-58) and a significant increase was also observed in the mRNA levels of this gene especially in mesenteric fat. The activity of HSL is also well known to be regulated posttranscriptionally by reversible phosphorylation and our study suggested a significant increase in HSL protein levels and activity (via phosphorylation) in both fat depots. Since a very recent study suggested that adipose tissue from mice exposed to a HFD present “obesity memory”, which means that present a tissue-autonomous lipolytic defect allowing increased efficiency of lipid storage^40^, synergistic approaches are needed to counteract such defects. Thus, the use of FMT emerges as a very positive and necessary approach along with a caloric restriction to reduce adiposity and obesity despite the “obesity memory” of the mesenteric and retroperitoneal adipocytes induced by the ingestion of a HFD.

Increased rates of lipolysis in mesenteric adipose tissue could be associated with fat liver accumulation^41^ and insulin resistance^42^. However, increasing lipolysis in adipose tissue does not necessarily increase serum free fatty acids (FFAs) levels because it could cause a shift within adipocytes or liver towards increased fatty acid utilization and, thus, protect against obesity. In line with this, our study has demonstrated that FMT in rodents reduces weight loss and fat mass through increased lipolysis but without developing fatty liver or increasing circulating FFA. Moreover, FMT also improves insulin resistance and this could be associated with FMT-induced fatty acid oxidation in liver, as suggested by our mRNA results.

Concerning gut microbiota composition at genera level, the ingestion of a HFD was accompanied by changes in several bacterial genera, whereas a moderate caloric restriction was able to counteract the changes induced by the HFD in the abundance of *Lactococcus, Anaerotruncus, Oscillospira, Streptococcus, Bilophila* and *Ruminococcus*. These results are in line with the study from Wang *et al*., (2018) that demonstrates that gut microbiota mediates the antiobesity effects of a caloric restriction^37^. However, FMT did not significantly modify gut microbiota composition compared to the CR and only significant increases in *Bifidobacterium* and *Blautia* genera were observed. Interestingly, the increase in both bacterial genera was only observed after the transplantations. *Bifidobacterium*, that belongs to *Actinomycetes* phylum, is one of the most numerous commensal bacteria present in mammalian gut. It helps *Bacteroides* degrade polysaccharides^43^ and inhibits exogenous cholesterol absorption from the small intestine^44^, although the beneficial effects on lipid metabolism and body weight could depend on the strain^45^. In general terms, *Bifidobacterium* has been suggested as a potential therapeutic candidate for management of obesity^45^; thus, the increase observed after FMT could mediate, at least in part, the lower body weight, adiposity and insulin resistance observed in these mice. *Blautia* is also a bacterial genera significantly and inversely associated with visceral fat accumulation in adults, regardless of gender^46^. In fact, different approaches that improve obesity and associated disorders such as laparoscopic sleeve gastrectomy or silybin, a naturally occurring hepatoprotective agent, have also demonstrated to increase the abundance of this genus ^47,48^. More studies are needed to deeply investigate the mechanisms involved in the potential association among *Bifidobacterium* and *Blautia* abundance and improvements in obesity. *Bifidobacteria* and *Bacteroides spp* are known to reinforce intestinal integrity^49^. Although we have not directly measured gut permeability, we quantified one marker of bacterial translocation in plasma (LBP, supplementary figure 5). Our results showed that HFD mice presented a significant increase in LBP plasma levels as expected, and CR was able to reduce such increase independently of the fecal transplantations. These results suggested that the beneficial effects observed after fecal transplantations are not secondary to improvements in bacterial translocation and, therefore, the health-promoting effects of *Bifidobacterium* seem to be independent to their actions on intestinal barrier function. In addition, the slight changes observed in gut microbiota composition after FMT could suggest that engraftment of bacterial microbiota is not needed to achieve such beneficial effects on obesity and adiposity in the short term and other components within the feces such as bacteriophages, or even nonliving components such as metabolites, could influence host energy homeostasis^16^. In fact, sterile fecal filtrates from resveratrol-fed mice have been demonstrated to be sufficient to improve glucose homeostasis in obese mice^50,51^. This issue deserves further investigation.

Antibiotic-treated animals are commonly used for FMT studies but problems with reproducibility, baseline values and antibiotic resistance genes should be considered^52^. Our study shows that all the positive effects observed after FMT disappeared if animals were previously treated with antibiotics for 6 days, which is in line with a previous study that demonstrated that antibiotic administration largely abolishes the metabolism-regulatory functions of gut microbiota and abrogated the health-beneficial effect of gut microbiota^37^. Thus, our results should be taken into account in the design of strategies to modify gut microbiota in the context of antibiotic resistance era.

To sum up, we have demonstrated that fecal transplantation and, especially, autologous fecal transplantation, potentiates the effects of a moderate energy restriction on weight loss and adiposity in the short term, possibly by decreasing feed efficiency and increasing adipose tissue lipolysis and, possibly, via increased hepatic fatty acid oxidation. Although autologous fecal transplantation produced a significant increase in bacterial richness/diversity, no significant changes were observed on gut microbiota composition at phyla and/or bacteria genera level and only slight changes were observed in the abundance of *Bifidobacterium* and *Blautia* genera, suggesting that other mechanisms different from changes in gut microbiota composition or bacterial microbiota engraftment participates in such beneficial effects. The additive effects of fecal transplantation and, especially, autologous procedure and energy restriction in obesity and its associated metabolic disorders could be very positive for those obese patients that do not respond well to moderate restrictive diets. In fact, these synergic effects could improve dietary adherence and could help to achieve the loss of weight needed to improve health with no need to follow more restrictive diets or to start other therapeutic/surgical options. These results could also reinforce the need for stool banking to deposit “lean” feces for later use.

## Materials and Methods

### Animal experiments

Forty-two male mice (C57BL/6 J) (5 weeks old) purchased from Charles River (Barcelona, Spain) were randomly assigned to the following groups: i) ***Control***: fed with a normal chow diet (Standard diet, 801010 RM1A (P), SDS, Essex, UK) for 18 weeks; ii) ***HFD***: animals fed with a HFD (60% of kcal from fat: D12492) (Research Diets Inc., New Brunswick, NJ, USA) for 18 weeks; iii) ***CR*** group: mice fed with a HFD for 12 weeks and 6 weeks under CR (−25% of daily calories). A 25% degree of energy restriction was chosen following the majority of studies carried out (ranging from 20 to 40% of energy restriction)^53,54^. These mice received water by oral gavage at the latter two weeks (weeks 17 and 18, once per week); iv) ***FT-H***: similar to the previous group. These animals received feces from control mice at the latter two weeks (weeks 17 & 18, once per week); v) ***FT-A***: similar to the previous group but with administration of their own feces before developing obesity (at the beginning of the experimental period) (Fig. 1A). The amount of diet provided to restricted animals (groups: CR, FT-H and FT-A) was calculated on the basis of spontaneous food intake (calories) quantified in HFD-fed animals. The CR group was also submitted to oral gavage (water-placebo); therefore, these mice were under the same stressful conditions than the FT-H and FT-A animals.

The first day of the experimental period (day 0) stools from control mice and FT-A animals were collected and frozen at −80 °C. At week 17 and week 18, 70 mg of the stools were hydrated in 250 µl of distilled water. Transplant into recipient mice was achieved by oral gavage of 152 µl of the supernatant obtained after centrifugation. Pooled stools from controls were used for transplantation in FT-H mice, whereas each mouse from the FT-A group received their own feces collected at day 0 following this procedure (Fig. 1A).

Antibiotic pretreatment is commonly used in clinical practice in FMT transplantation for *C. difficile* infection^19^ and also in studies of FMT in rodents^52^. Thus, in order to check if antibiotic pretreatment potentially increase the response of transplants on body weight gain and adiposity, another three groups of mice were included in the design of the experiment. These groups were similar to CR, FT-H and FT-A mice but treated with antibiotics for 6 days before performing the first fecal transplantation (1 mg/ml ampicillin and 1 mg/ml neomycin in the drinking water) (ampicillin provided by Normon, Madrid, Spain and neomycin provided by Sigma, St. Louis, MO, USA).

All mice were euthanized after 18 weeks of treatment and samples were collected after a 5 h fasting period. White adipose tissue (WAT) from different anatomical locations (mesenteric, epidydimal, retroperitoneal and subcutaneous), liver, muscle and other organs were dissected, weighed and immediately frozen in liquid nitrogen or fixed in 10% formalin and paraffin embedded for subsequent histological studies. Stools were collected at the beginning of the project (time point 0; T0), 24 hours before the first transplant (time point 1; T1), 24 hours after the first transplant (time point 2; T2), 24 hours after the second transplant (time point 3; T3) and at the end of the experimental period (time point 4; T4) (Fig. 1A). All procedures were carried out in accordance with the European Communities Council Directive on animal experiments (EU Directive 2010/63/EU) and were approved by the Institutional Animal Care & Use Committee (IACUC) from the Center for Biomedical Research of La Rioja, Spain (CIBIR).

### Biochemical parameters and Bacterial Translocation

Serum samples were collected from cardiac puncture after 5 hours of fasting. Levels of glucose, triglycerides, aspartate aminotransferase (AST) and alanine aminotransferase (ALT) were measured using an automatic biochemical analyzer (Cobas C711, Madrid, Spain). Insulin was quantified by a commercial ELISA Kit following manufacturer’s instructions (EMD Millipore, MO, USA). Insulin resistance was calculated using the homeostasis model assessment of insulin resistance (HOMA-IR) as previously described^55^. Plasma levels of lipopolysaccharide binding protein (LBP) were measured using an ELISA from Hycult Biotech (Uden, The Netherlands)^56^.

### Histological analyses

Following formalin fixation, adipose tissue from different fat depots (mesenteric, retroperitoneal and epididymal) were dehydrated and paraffin embedded. Tissue sections (5 μm-thick) were rehydrated and stained with hematoxylin-eosin (H&E) according to standard protocols. The fields were evaluated with the final magnification of 40×. Digital photographs were taken from each histological section and the number of adipocytes and their size was quantified using automatized software (Image-J Software).

### Real-time gene expression analysis

To evaluate gene expression in liver, mesenteric and retroperitoneal fat depots, the same protocol than previously described was followed^57^. In brief, total RNA was extracted using TRIzol reagent (Invitrogen, Carlsbad, CA, USA) according to the manufacturer’s instructions. RNA concentration and quality was evaluated using a Nanodrop Spectrophotometer (ND-1000, Thermo Fisher Scientific, MA, USA). A 2 μg sample of total RNA was incubated with DNase (DNAse I Amplification Grade, Invitrogen, MA, USA) for 30 min at 37 °C. RNA was then reverse-transcribed to cDNA using MMLV reverse transcriptase (Invitrogen, Carlsbad, CA, USA). Sybr Premix Ex Taq (Takara Bio Inc., Shiga, Japan) and specific primers for comparative gene identification 58 (CGI58), peroxisome proliferator-activated receptor alpha (PPARalpha), peroxisomal acyl-coenzyme A oxidase 1 (ACOX), carnitine palmitoyltransferase 1A (CPT1A) and glucose transporter 4 (GLUT4) were used for quantitative real-time PCR (qRT-PCR) (Supplementary Table 2) (all from Sigma, St. Louis, MO, USA).

All procedures were performed according to the manufacturer’s instructions using the ABI PRISM 7300 (Applied Biosystems, Foster City, CA, USA). All PCR reactions were performed in triplicate, and actin was used to normalize gene expression. Ct values were generated by the ABI software. Finally, the relative expression level of each gene was calculated as foldchange: 2^−ΔΔCt^^58^.

### Western-blot analysis

Western blot analyses were performed in mesenteric and retroperitoneal fat depots. Lysates were obtained by the addition of a RIPA buffer containing protease and phosphatase inhibitors (Roche/Sigma, St. Louis, MO, USA) and homogenization with a Pellet Pestle. Protein extracts were collected after sample centrifugation. Proteins were quantified with the BCA method according to the supplier’s instructions (Pierce-Thermo Scientific, Rockford, IL, USA). 20–45 ug of total proteins were denatured and resolved in SDS-PAGE mini-gels and electroblotted onto 0.2um nitrocellulose membranes (Trans-Blot Turbo Transfer Pack, Bio-Rad, Hercules, CA, USA). The membranes were stained with Ponceau S to confirm equal sample loading, and then, blocked and incubated with specific antibodies against ATGL, HSL, HSL phospho Ser^660^, HSL phospho Ser^563^ and HSL phospho Ser^565.^ (all from Cell Signaling Technologies, Beverly, MA, USA, Supplementary Table 3). Secondary antibody was anti-rabbit IgG-HRP (Cell Signalling Technologies, Beverly, MA, USA). The immunoreactive proteins were detected with highly sensitive chemiluminescent detection reagent (ECL Prime Western Blotting Detection Reagent, Amersham, GE Healthcare Life Sciences, Pittsburgh, PA, USA). Band intensities were quantified using the Image-J Software and normalized to the band densities of the Ponceau S staining (Fluka Analytical/Sigma, St. Louis, MO, USA).

### Gut microbiota: DNA extraction from stool samples and 16 S rRNA gene sequencing

Gut microbiota study was performed following the same protocol than previously described with slight modifications^59^. Thus, fresh stool samples were collected from all animals at different time points and frozen at −80 °C (Fig. 1A). Faecal DNA was extracted from 10 mg of stools using the QIAGEN DNeasy Blood & Tissue Kit (Qiagen, Venlo, The Netherlands) and purity and concentration were subsequently determined by Qubit 3.0 fluorometer (Thermo Fisher Scientific, MA, USA). Sequencing was carried out by an Illumina sequencer (MiSeq, 2×300 pb, paired-end) (Illumina Inc., San Diego, CA, USA). FastQC and Trim Galore programs were used for quality control and adapter trimming (https://www.bioinformatics.babraham.ac.uk/projects/fastqc/)(https://www.bioinformatics.babraham.ac.uk/projects/trim_galore/). Reconstruction of full-length V3-V4 16 S rRNA gene regions for taxonomic assignment and the determination of operational taxonomic units (OTUs) were carried out through the QIIME program (v1.9.1) using the Greengenes database. Uclust program was used for the establishment of taxonomy clusters (http://drive5.com/usearch/manual/uclust_algo.html). The MicrobiomeAnalyst website was used for the statistical analysis of metagenomics that includes the evaluation of α and β-diversity as well as the differential abundances among the groups^59,60^. The measure of sample-level species richness was analyzed using observed species, Chao-1 and Simpson indexes. Differential abundances among the groups were calculated using Univariate analyses at Phylum and Genera taxonomic levels. In order to analyze the temporal dynamics of detected taxononomic groups at the Phylum and Genera levels in our dataset, the MetaLonDA R package (v1.1.5) was used to perform pairwise comparisons over selected groups^61^. The MetaLonDA R-package employs a negative binomial distribution in conjunction with a standard Smoothing Spline ANOVA (SS-ANOVA) approach to model the read counts. Then, it performs the significance testing based on unit time intervals by using permutation testing procedure. The software fits a curve for each phenotypic/treatment group and compares the area between the two curves.

### Statistical analysis

Statistical analysis of metagenomics data was performed using the web-tool *MicrobiomeAnalyst*. Differential abundance analysis (comparisons among groups) was carried out by classical univariate analysis using Kruskal-Wallis or Mann-Whitney U tests; P value <0.05 following a false discovery rate (FDR) correction for multiple comparisons was considered statistically significant. The Benjamini-Hochberg approach was used for FDR calculation. Data obtained from α-diversity were statistically analyzed by Kruskal-Wallis or Mann-Whitney U tests while β-diversity was statistically analyzed using the Wilcoxon rank-sum non-parametric test. A Principal Coordinate Analysis (PCoA) was also developed. Results were plotted according to the first two principle components.

Results are presented as mean ± standard error of the mean. Comparisons among groups were performed with unpaired t test/Mann Whitney U-test or ANOVA/Kruskall Wallis depending on the normality of the data (calculated with Shapiro-Wilk test). Statistical analysis was carried out using SPSS 19.0 (SPSS Inc., Chicago, IL, USA) and GraphPad Prism 6 (GraphPad Prism, La Jolla, CA, USA). P values <0.05 were considered statistically significant.

## Supplementary information

Supplementary Information.
