# Supplementary material for: Autologous fecal transplantation from a lean state potentiates caloric restriction effects on body weight and adiposity in obese mice
Source: Sci Rep. 2020 Jun 10;10:9388. doi: 10.1038/s41598-020-64961-x (PMC7287061; doi:10.1038/s41598-020-64961-x)
Supplement: Supplementary file 1 — Supplementary Information. [file 41598_2020_64961_MOESM1_ESM.doc]

**Autologous fecal transplantation potentiates caloric restriction effects on body weight and adiposity**

Patricia Pérez-Matute^1^*, María Íñiguez^1^, María de Toro^2^, Emma Recio-Fernández^1^ and José A. Oteo^1,2^

^1^Infectious Diseases, Microbiota and Metabolism Unit. Infectious Diseases Department, Center for Biomedical Research of La Rioja (CIBIR). Logroño, (La Rioja), Spain.

^2^Genomics & Bioinformatics Core Facility. CIBIR. Logroño, La Rioja

^3^Infectious Diseases Department. Hospital Universitario San Pedro, Logroño, (La Rioja), Spain.

Patricia Pérez Matute: [cpperez@riojasalud.es](mailto:cpperez@riojasalud.es)

María Íñiguez: miniguez@riojasalud.es

María de Toro: mthernando@riojasalud.es

Emma Recio-Fernández: ereciof@riojasalud.es

José A. Oteo: jaoteo@riojasalud.es

**Corresponding author:**

Dr. Patricia Pérez-Matute: [cpperez@riojasalud.es](mailto:cpperez@riojasalud.es)

Infectious Diseases, Microbiota and Metabolism Unit

Infectious Diseases Department

Center for Biomedical Research of La Rioja (CIBIR)

Piqueras 98, 26006 Logroño, La Rioja, Spain.

Tl. (+34) 941 278 867 ext. 84871

FAX. (+34) 941 278 887

**Abbreviated title: Fecal Autotransplant and Obesity**

**Supplementary Table 1**

|  |  | **Control** | **HFD** | **CR** | **FT-H** | **FT-A** | **Kruskal-Wallis test P value** |
| --- | --- | --- | --- | --- | --- | --- | --- |
| **Total experimental period**  **(18 weeks)** | **Food intake (g/day)** | 3.63 ± 0.03 | 2.85 ± 0.03 | 2.36 ± 0.00***^#^ | 2.39 ± 0.01*** | 2.38 ± 0.01***^#^ | < 0.0001 |
|  | **Calorie intake (g/day)** | 11.25 ± 0.10 | 14.57 ± 0.16**** | 12.06 ± 0.00^#^ | 12.22 ± 0.08 | 12.18 ± 0.09^#^ | < 0.0001 |
| **Caloric restriction period**  **(6 weeks)** | **Food intake (g/day)** | 3.57 ± 0.06 | 2.94 ± 0.06 | 2.12 ± 0.00***^#^ | 2.12 ± 0.00****^##^ | 2.13 ±0.00** | < 0.0001 |
|  | **Calorie intake (cal/day)** | 11.08 ± 0.19 | 15.03 ± 0.35** | 10.81 ± 0.02^##^ | 10.85 ± 0.00^###^ | 10.87 ± 0.00^#^ | < 0.0001 |

***p*<0.01, ****p*<0.001, *****p*<0.0001 *vs* Control; ^#^*p*<0.05, ^##^*p*<0.01, ^###^*p*<0.001 *vs* HFD according to Kruskal-Wallis test followed by post hoc multiple comparisons by Dunn´smultiple comparisons test.

**Supplementary Table 2**: Primers sequences used for real-time-PCR

| **Gene** | **Metabolic pathway** | **Forward** | **Reverse** |
| --- | --- | --- | --- |
| **CGI58** | Lipolysis | TGACAGTGATGCGGAAGAAG | AGATCTGGTCGCTCAGGAAA |
| **PPARα** | FA β-oxidation | GAGGGTTGAGCTCAGTCAGG | GGTCACCTACGAGTGGCATT |
| **ACOX** | FA β-oxidation | CAGGAAGAGCAAGGAAGTGG | CCTTTCTGGCTGATCCCATA |
| **CPT1α** | FA β-oxidation | CCAGGCTACAGTGGGACATT | GAACTTGCCCATGTCCTTGT |
| **GLUT4** | Glucose transporter, insulin-responsive | ACTCTTGCCACACAGGCTCT | AATGGAGACTGATGCGCTCT |
| **β-actin** | Housekeeping gene | GGCTGTATTCCCCTCCATCG | CCAGTTGGTAACAATGCCATGT |

FA: fatty acid

**Supplementary Table 3**: List of primary antibodies used in Western Blotting analysis.

| **Antigen** | **Manufacturer** | **Catalog Number** | **Source** |
| --- | --- | --- | --- |
| **ATGL** | Cell Signaling Technology | 2439S | Rabbit |
| **HSL** | Cell Signaling Technology | 4107S | Rabbit |
| **Phospho-HSL (Ser660)** | Cell Signaling Technology | 4126S | Rabbit |
| **Phospho-HSL (Ser563)** | Cell Signaling Technology | 4139S | Rabbit |
| **Phospho-HSL (Ser565)** | Cell Signaling Technology | 4137S | Rabbit |

**Supplementary Figure 1: Body weight and adipose tissue alterations upon CR and FMT in antibiotic-treated animals.** (**A**) Body weight gain of control and HFD mice with and without FMT with/without antibiotic treatment for 6 days. (**B**) Effects of CR and FMT on mesenteric and retroperitoneal fat weights in control and HFD-induced obese mice under CR and/or FMT with/without antibiotic treatment. Data are expressed as mean ± SEM of at least 8 animals per group. **p*<0.05, ***p*<0.01, ****p*<0.001, *****p*<0.0001 *vs* Control; ^#^*p*<0.05, ^##^*p*<0.01, ^###^*p*<0.001, ^####^*p*<0.0001 *vs* HFD, *^a^p*<0.05 *vs* CR (placebo group respectively), %*p*<0.05 *vs* FT-A with no antibiotic exposure.

**Supplementary Figure 2: Liver weight and transaminases serum levels upon CR and FMT.** (**A**) Liver weight gain of control and HFD mice with and without fecal transplantation. (**B**) Effects of CR and fecal transplantation on serum GOT and GPT levels in control and HFD-induced obese mice. Data are expressed as mean ± SEM of at least 8 animals per group. **p*<0.05, ****p*<0.001 *vs* Control; ^#^*p*<0.05, ^##^*p*<0.01, ^###^*p*<0.001, *vs* HFD.

**Supplementary Figure 3: Relative abundance of *Bifidobacterium* and *Blautia* genera present in mice under moderate caloric restriction (CR) and after autologous fecal transplantation (FT-A).** Counts are showed log-transformed.

**Supplementary Figure 4: Principal Coordinate Analysis (PCoA).** (**A**) PCoA at the beginning of the study. (**B**) PCoA 24 hours prior first transplantation was carried out (T1). (**C**) PCoA at the end of the experimental period (after two transplants were carried out).

**Supplementary Figure 5: Bacterial Translocation in control and HFD mice with and without FMT.** LBP, lipopolysaccharide-binding protein. Data are expressed as mean ± SEM of at least 8 animals per group. **p*<0.05 *vs* Control; ^#^*p*<0.05, ^##^*p*<0.01,*vs* HFD.

**Supplementary Figure 6: Full length blots from total ATGL protein levels in mesenteric (A) and retroperitoneal (B) adipose tissues.** The region cropped and presented in figure 2 has been indicated in the corresponding full-length gel with a red rectangle.

**Supplementary Figure 7: Full length blots from total HSL protein levels in mesenteric (A) and retroperitoneal (B) adipose tissues.** The region cropped and presented in figure 2 has been indicated in the corresponding full-length gel with a red rectangle.

**Supplementary Figure 8: Full length blots from HSL phospho Ser^563^ protein levels in mesenteric (A) and retroperitoneal (B) adipose tissues.** The region cropped and presented in figure 2 has been indicated in the corresponding full-length gel with a red rectangle.

**Supplementary Figure 9: Full length blots from HSL phospho Ser^660^** **protein levels in mesenteric (A) and retroperitoneal (B) adipose tissues.** The region cropped and presented in figure 2 has been indicated in the corresponding full-length gel with a red rectangle.

**Supplementary Figure 10: Full length blots from Ponceau Staining in mesenteric (A) and retroperitoneal (B) adipose tissues.** The region cropped and presented in figure 2 has been indicated in the corresponding full-length gel with a red rectangle.

**Supplementary Figure 1**


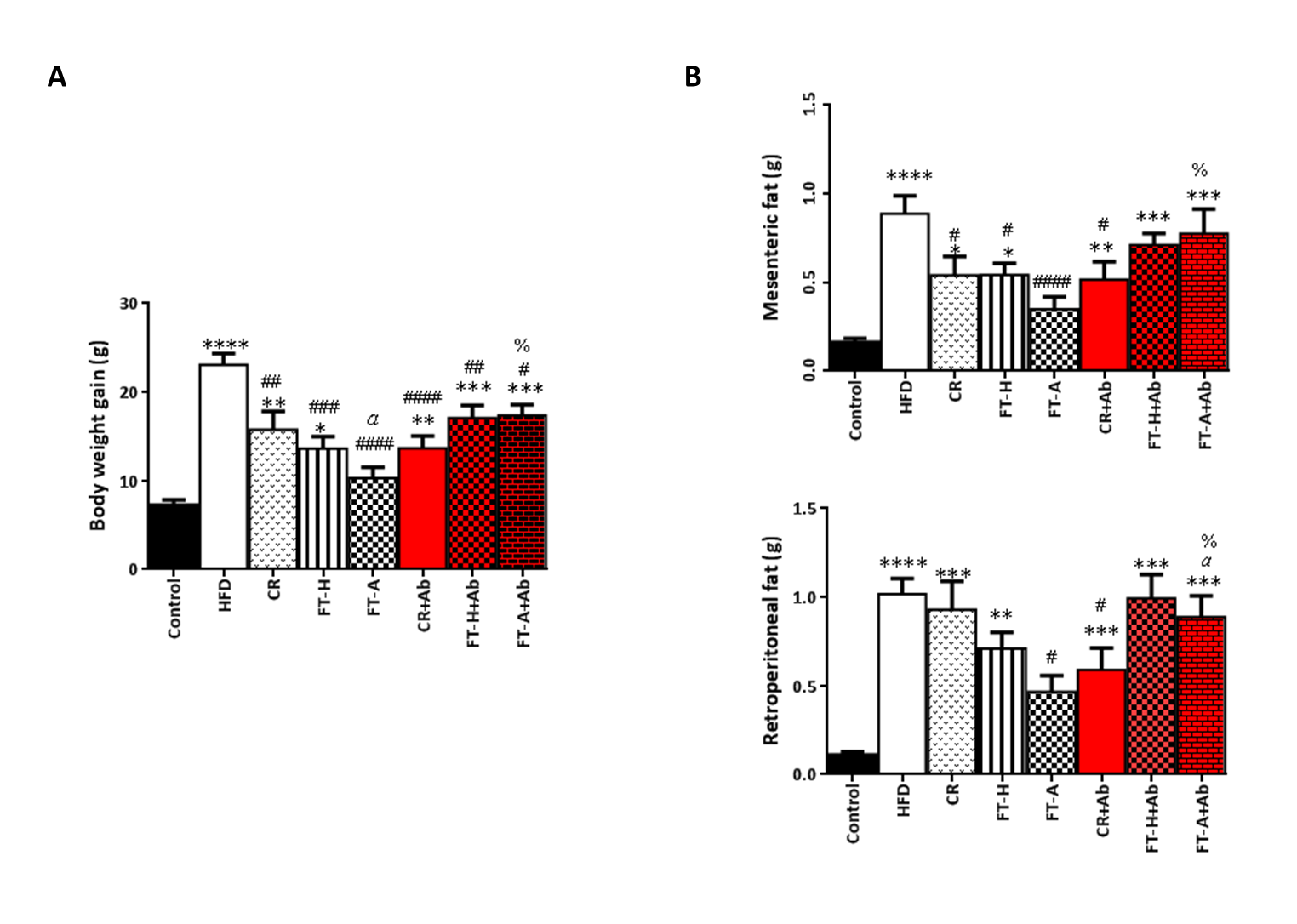


**Supplementary Figure 2**


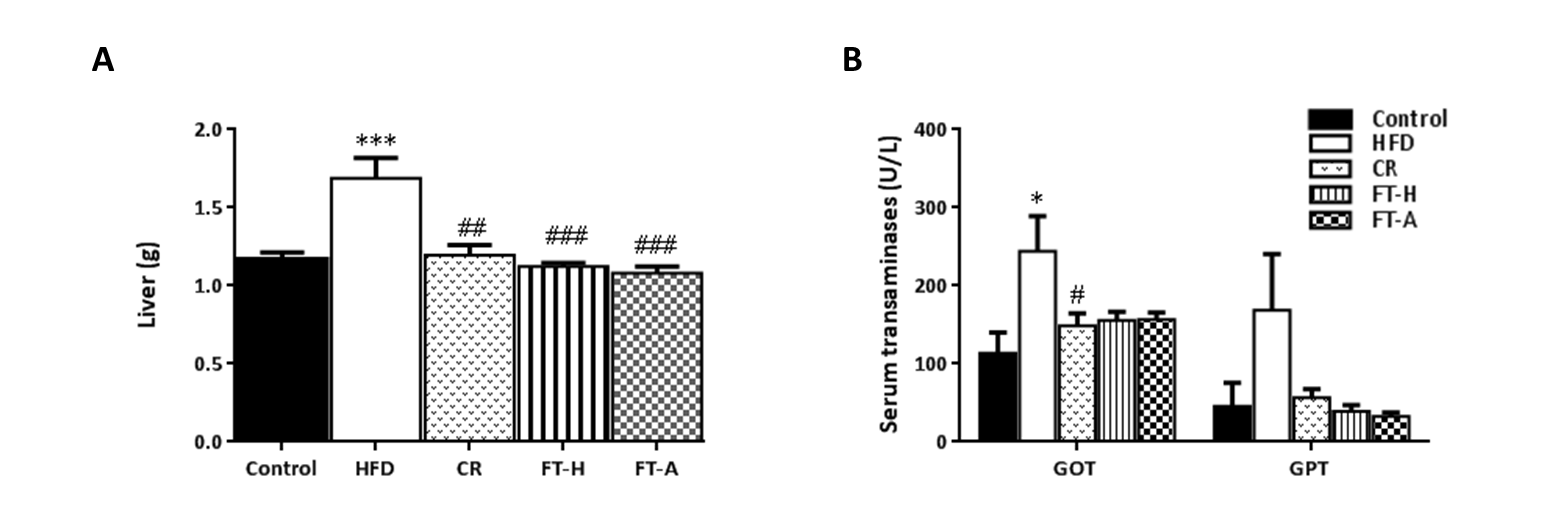


**Suplementary Figure 3**


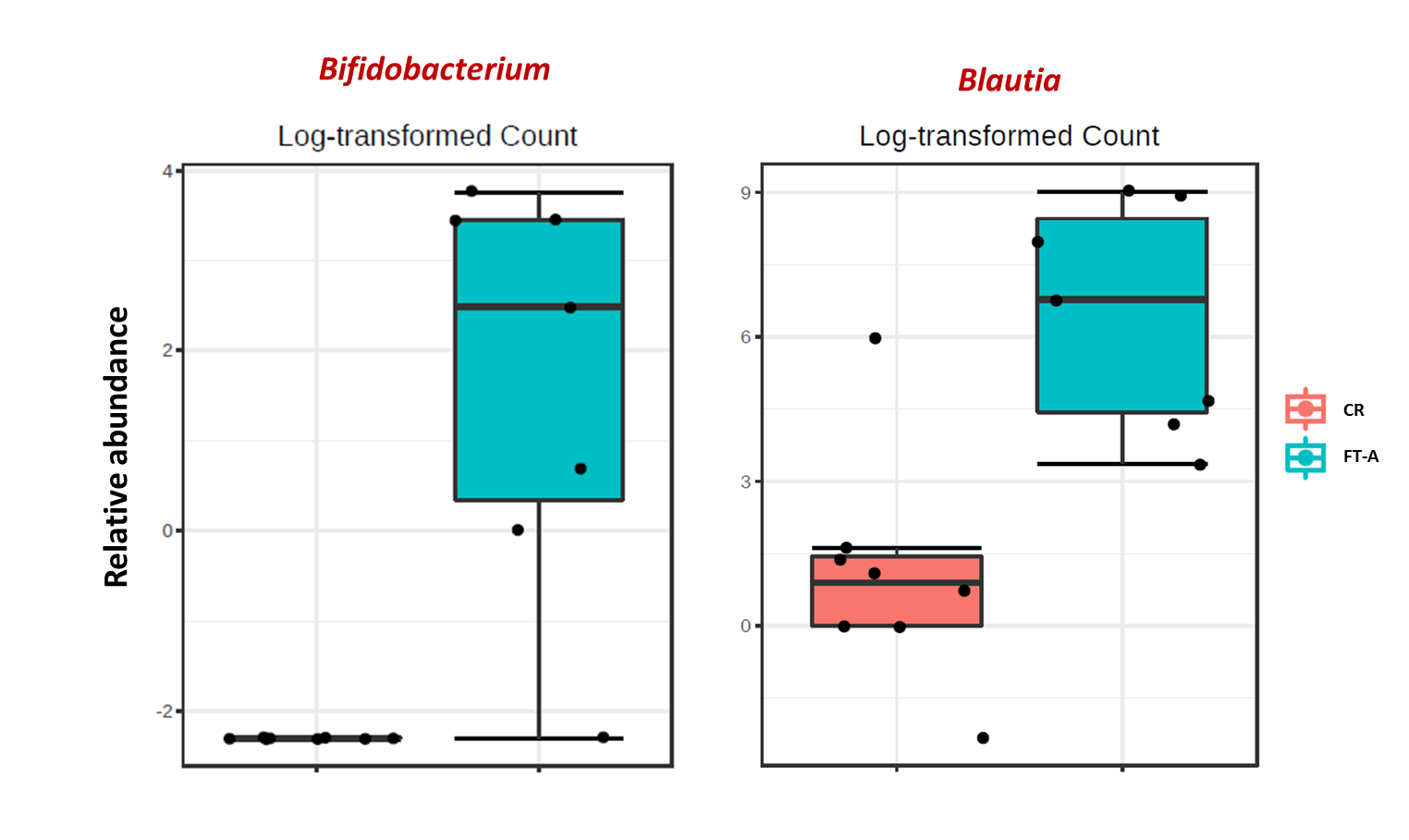


**Suplementary Figure 4:**


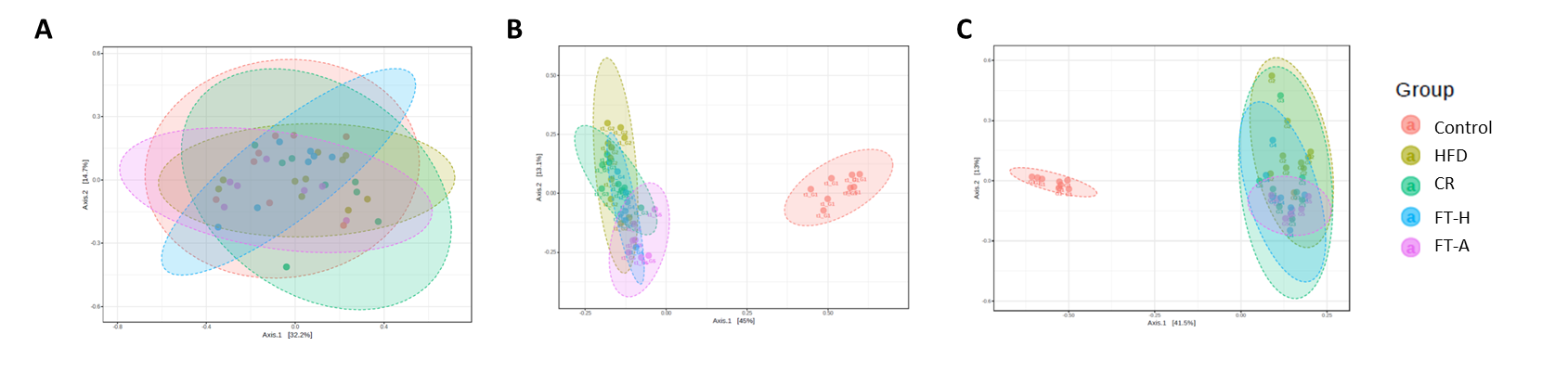


**Supplementary Figure 5**

**Supplementary 6:**

**
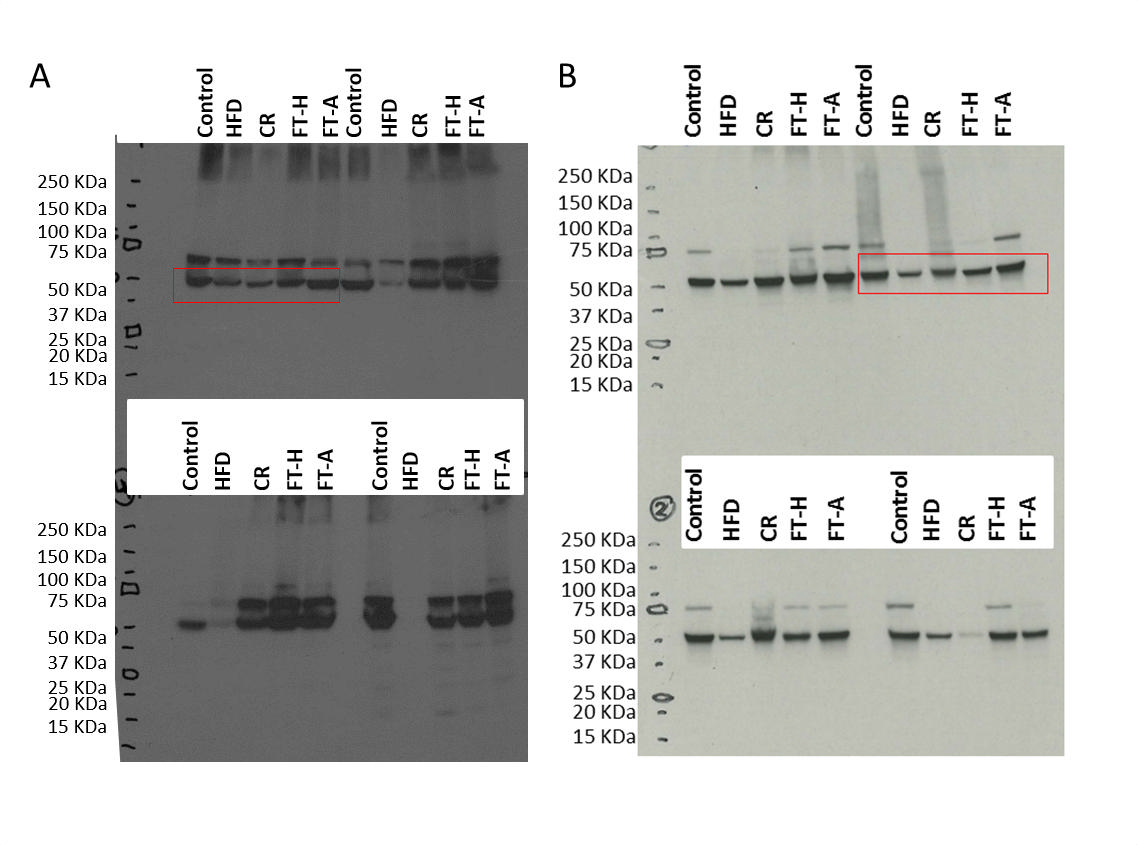
**

**Supplementary figure 7**

**
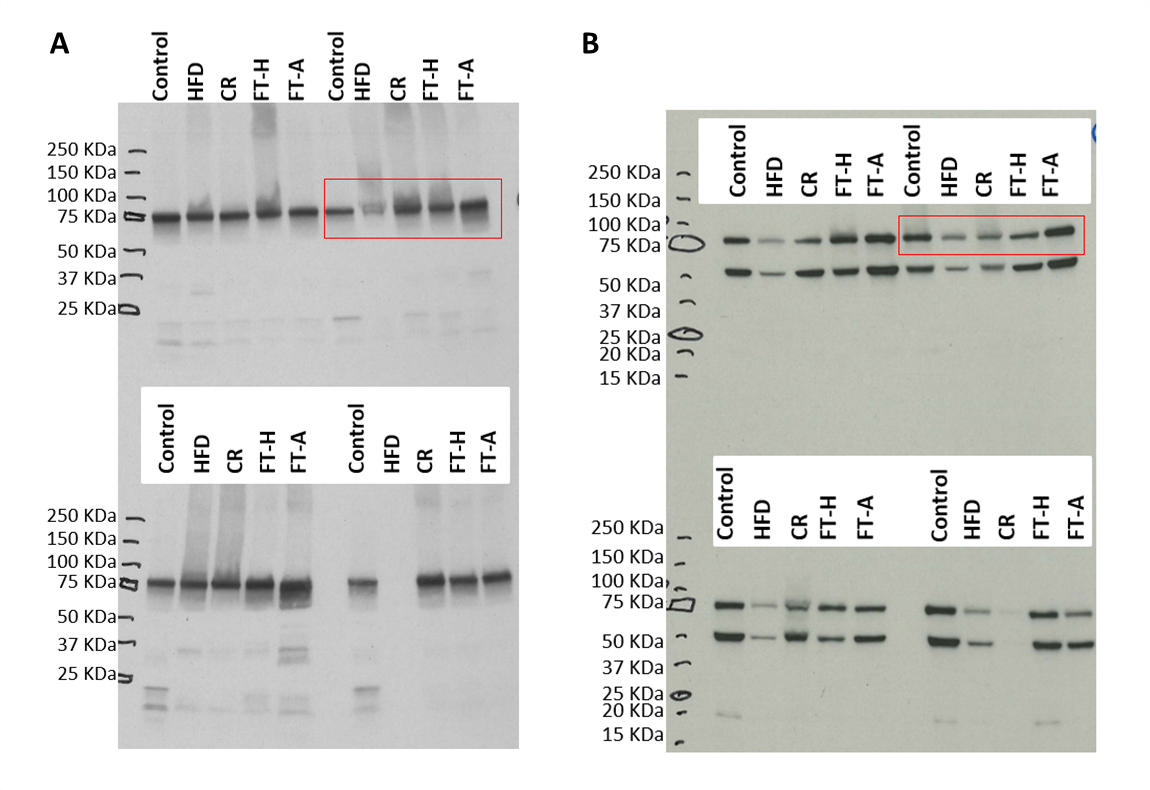
**

**Supplementary figure 8**

**
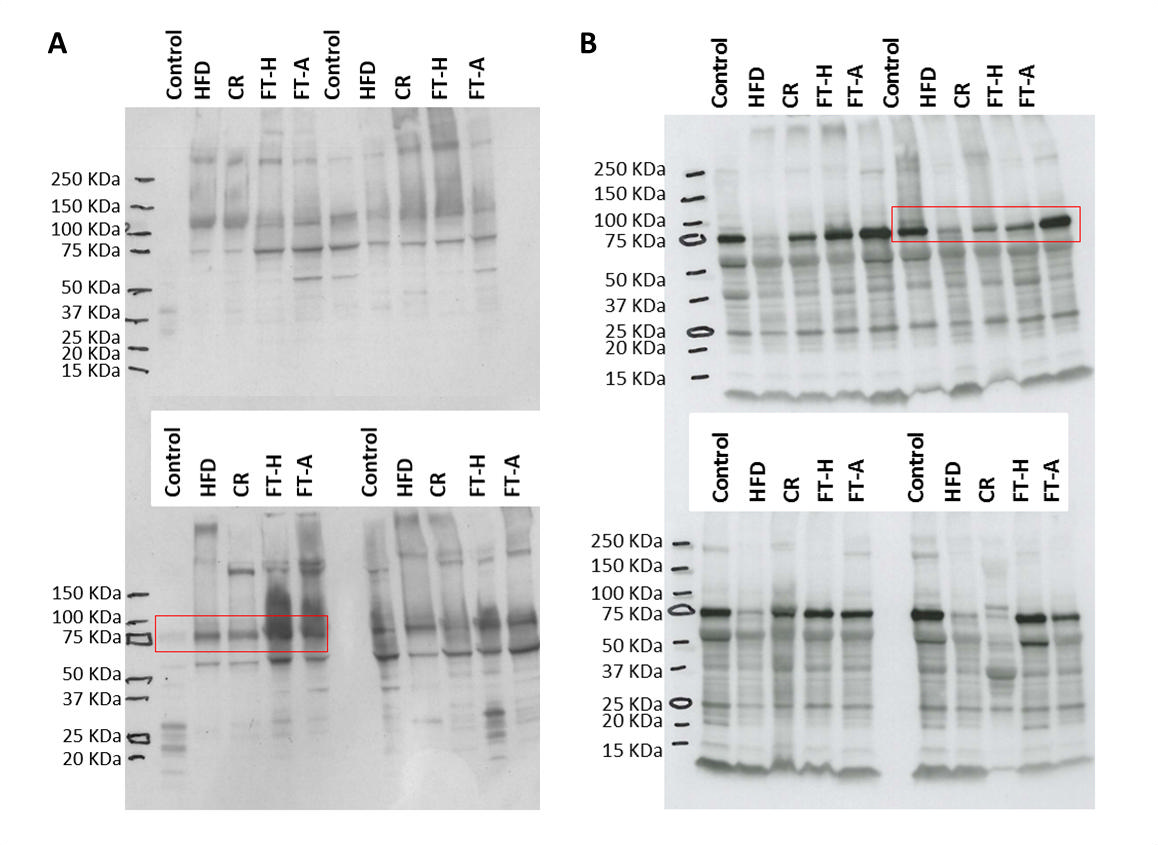
**

**Supplementary figure 9:**

**
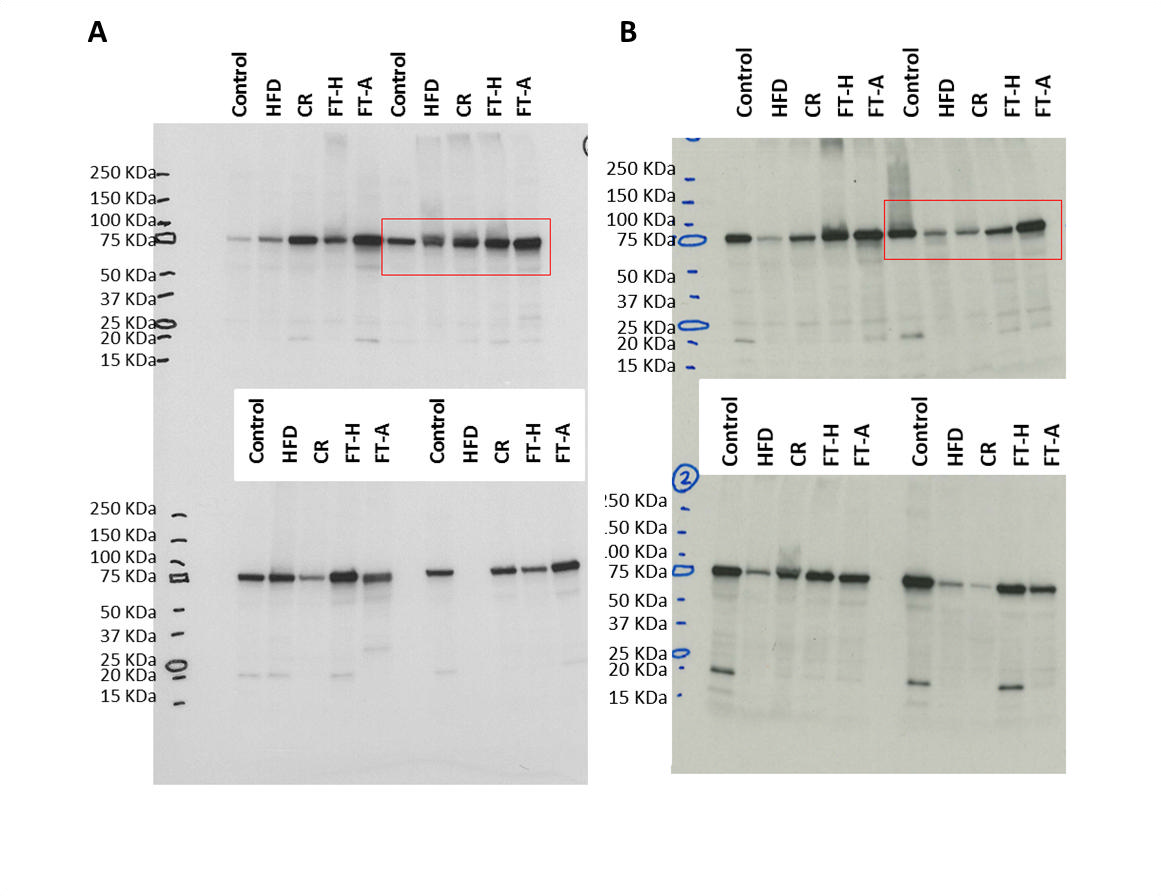
**

**Supplementary figure 10:**

**
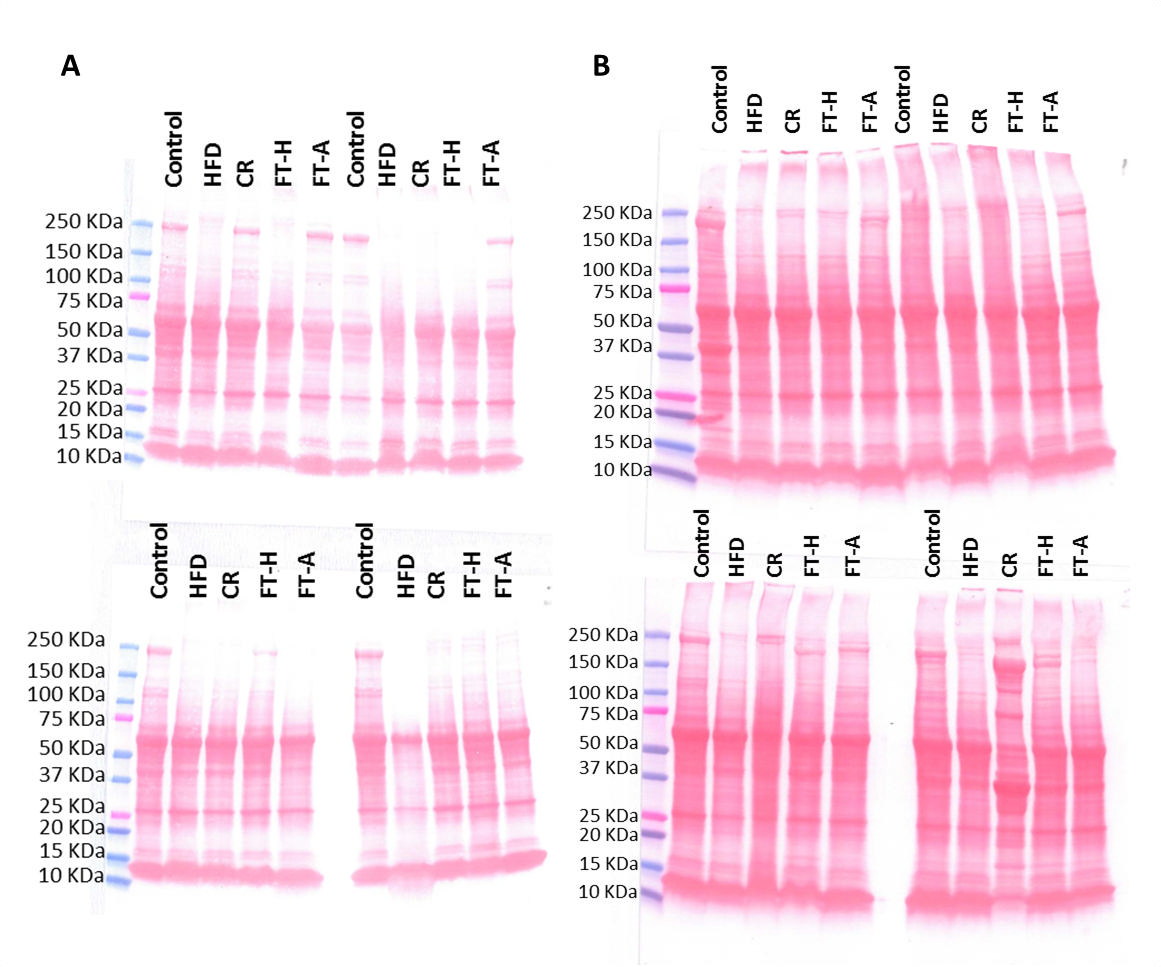
**
